# Assessment of mouse-specific pharmacokinetics in kidneys based on ^131^I activity measurements using micro-SPECT

**DOI:** 10.1186/s40658-022-00443-5

**Published:** 2022-02-23

**Authors:** Clarita Saldarriaga Vargas, Lara Struelens, Matthias D’Huyvetter, Vicky Caveliers, Peter Covens

**Affiliations:** 1 Radiation Protection Dosimetry and Calibrations, Belgian Nuclear Research Centre (SCK CEN), Boeretang 200, 2400 Mol, Belgium; 2 Laboratory for In Vivo Cellular and Molecular Imaging, Department of Medical Imaging, Vrije Universiteit Brussel, Brussels, Belgium

**Keywords:** Micro-SPECT, Activity quantification, Accuracy, Kidney, Mouse-specific pharmacokinetics, Single-domain antibody fragment, HER2

## Abstract

**Background:**

In order to acquire accurate drug pharmacokinetic information, which is required for tissue dosimetry, micro-SPECT must be quantitative to allow for an accurate assessment of radioligand activity in the relevant tissue. This study investigates the feasibility of deriving accurate mouse-specific time-integrated drug pharmacokinetic data in mouse kidneys from activity measurements using micro-SPECT.

**Methods:**

An animal experiment was carried out to evaluate the accuracy of ^131^I activity quantification in mouse kidneys (mean tissue volume of 0.140 mL) using a micro-SPECT system against conventional ex vivo gamma counting (GC) in a NaI(Tl) detector. The imaging setting investigated was that of the mouse biodistribution of a ^131^I-labelled single-domain antibody fragment (sdAb), currently being investigated for targeted radionuclide therapy of HER2-expressing cancer. SPECT imaging of ^131^I 365-keV photons was done with a VECTor/CT system (MILabs, Netherlands) using a high-energy mouse collimator with 1.6-mm-diameter pinholes. For both activity quantification techniques, the pharmacokinetic profile of the radioligand from approximately 1–73 h p.i. was derived and the time-integrated activity coefficient per gram of tissue (*ã*/*M*) was estimated. Additionally, SPECT activity recovery coefficients were determined in a phantom setting.

**Results:**

SPECT activities underestimate the reference activities by an amount that is dependent on the ^131^I activity concentration in the kidney, and thus on the time point of the pharmacokinetic profile. This underestimation is around − 12% at 1.5 h (2.89 MBq mL^−1^ mean reference activity concentration), − 13% at 6.6 h (149 kBq mL^−1^), − 40% at 24 h (17.6 kBq mL^−1^) and − 46% at 73 h (5.2 kBq mL^−1^) p.i. The *ã*/*M* value estimated from SPECT activities is, nevertheless, within − 14% from the reference (GC) *ã*/*M* value. Furthermore, better quantitative accuracy (within 2% from GC) in the SPECT *ã*/*M* value is achieved when SPECT activities are compensated for partial recovery with a phantom-based recovery coefficient of 0.85.

**Conclusion:**

The SPECT imaging system used, together with a robust activity quantification methodology, allows an accurate estimation of time-integrated pharmacokinetic information of the ^131^I-labelled sdAb in mouse kidneys. This opens the possibility to perform mouse-specific kidney-tissue dosimetry based on pharmacokinetic data acquired in vivo on the same mice used in nephrotoxicity studies.

**Supplementary Information:**

The online version contains supplementary material available at 10.1186/s40658-022-00443-5.

## Introduction

Pharmacokinetic data derived from preclinical studies in in vivo biological models play a key role in the prediction of radiation dosimetry of first-in-human studies of targeted radionuclide therapy. Accurate quantification of radioligand activity in preclinical radiobiological studies is necessary for a sound investigation of the preclinical absorbed dose to normal (healthy) animal tissues.

Targeted radionuclide therapy with radioligands based on small targeting molecules, such as peptides and antibody fragments (which typically show a significant retention in kidney tissue), can pose a concern in terms of radiation-induced nephrotoxicity. Consequently, the kidneys are often the subject of pharmacokinetic and dose-escalation studies during the preclinical testing of (new) radioligands.

Over the past decades, small-animal single-photon emission computed tomography (micro-SPECT) has seen significant improvements in spatial and temporal resolution, sensitivity and quantitative capability, leading to a growing use of this technique for quantitative imaging studies of drug pharmacokinetics in basic and (back)translational research [1–3]. Conventional radioactivity measurements of dissected (ex vivo) tissues via gamma counting (GC) require the sacrifice of many animals at multiple time points to ensure adequate sampling of drug pharmacokinetics. As opposed to GC, micro-SPECT offers the possibility to quantify radioactivity in tissues in vivo, allowing to derive pharmacokinetic information from one and the same animal. This eliminates the inter-variability effects in the assessment of the time dependence of drug pharmacokinetics, since each animal acts as its own control for different sampling time points. Moreover, in vivo micro-SPECT enables the possibility to perform mouse-specific dosimetry on animals used in (long-term) radiobiological studies of therapeutic response and toxicity.

In order to obtain accurate drug pharmacokinetic information, micro-SPECT must be quantitative and allow an accurate assessment of radioactivity in the relevant tissue. Similar to clinical SPECT, however, the accuracy of micro-SPECT images can be influenced by several factors. These include scatter and non-uniform attenuation of photons in tissue [4], partial volume effects (PVE) due to the limited spatial resolution of the imaging system [4], statistical and bias effects associated with the image reconstruction methods in relation with some imaging conditions (e.g. low-count studies, poor signal-to-noise ratio) [5, 6], artefacts resulting from the use of resolution modelling [7], etc. [8]. While methods to compensate for photon Compton scatter and attenuation are available and have been adopted in micro-SPECT [9, 10], the other effects strongly depend on the specific activity distribution as well as on the imaging and reconstruction settings used, and remain difficult to accurately compensate for in an automated fashion for any imaging setting [11]. An approach to compensate for the bias in SPECT activity recovery in a specific setting consists of employing an activity recovery coefficient (RC), defined as the ratio of the measured and true activity concentrations for a calibration object [12]. Measurements of physical phantoms with geometric shapes such as rods or spheres of varying sizes are used to determine RC for similarly sized anatomic structures. Although the use of this empiric approach has been increasing in clinical theranostics studies which rely on quantitative SPECT [12], it is usually not considered in current preclinical quantitative micro-SPECT studies. In addition to SPECT image-related factors, the accuracy of SPECT-based activity quantification can also be affected by other factors such as delineation of the volume of interest (VOI), activity calibration and post-reconstruction filtration. These aspects should also be considered carefully in the design and reporting of small-animal quantitative SPECT studies [13, 14].

This study evaluates the accuracy of Iodine-131 (^131^I) activity quantification in mouse kidneys using a micro-SPECT/CT imaging system and investigates the feasibility of this approach to derive mouse-specific time-integrated pharmacokinetic information as input for radiation dosimetry. The imaging setting investigated is that of the mouse biodistribution of the single-domain antibody fragment (sdAb) 2Rs15d radiolabelled with ^131^I, a radioligand previously reported and described in [15] which is currently being tested in humans for targeted radionuclide therapy of HER2-expressing cancer [16]. Considering the relevant retention of this sdAb in kidneys, an investigation on the accuracy of micro-SPECT imaging for assessing pharmacokinetics in mouse kidneys is pertinent to support (back)translational investigations on the dose—response of potential nephrotoxicity and radioprotective strategies. Additionally, a phantom study is performed to investigate the influence of the number of photon counts used for image reconstruction on SPECT activity recovery. The use of phantom-based recovery coefficients for improving the accuracy of micro-SPECT-based activity quantification is also investigated.

## Materials and methods

### SPECT-CT imaging

SPECT-CT acquisitions were performed with a VECTor/CT small-animal SPECT-CT system (U-PET/SPECT4, CT Skyscan1178; MILabs, Utrecht, Netherlands) [17]. For photon detection, the SPECT module uses three stationary large NaI(Tl) detectors each with a thickness of 18 mm. A high-energy mouse collimator with 114 pinholes of 1.6 mm diameter was used for imaging of 365-keV photons of ^131^I. The central field of view of the collimator (volume region observed by all pinholes simultaneously, in one bed position) has a diameter of approximately 12 mm and a longitudinal length of 9 mm [17].

All SPECT and CT images were generated and co-registered with VECTor’s manufacturer-provided software. SPECT images with 0.6-mm-wide cubic voxels were reconstructed using a 20% photo-peak window centred at 365 keV, using VECTor’s pixel-based ordered-subset expectation maximization (OSEM) iterative reconstruction algorithm [18]. Thirty iterations with two subsets (i.e. 60 OSEM updates, equivalent to 60 maximum-likelihood expectation–maximization iterations) were performed for all scans. A prior investigation indicated that 60 OSEM updates were sufficient to ensure convergence in the recovery of hot rods with a diameter in the range of 4 to 6 mm, which we consider to be comparable to the size of mouse kidneys. A system response matrix optimized for 364 keV photons was used [19]. Two adjacent background-and-scatter windows of approximately 6% width were used for Compton photon scatter and background signal correction using the triple-energy-window method [9]. Additionally, SPECT images were registered to CT images and the resulting SPECT images were corrected for photon attenuation based on CT data [10]. No post-reconstruction filters (for smoothing) were used. The final resampled SPECT images used for analysis have nearly cubic voxels of approximately 0.17 mm width.

SPECT voxel data (count rate, in reconstructed “cps”) were calibrated in terms of activity concentration (MBq mL^−1^) using a calibration factor determined from the SPECT scan of a syringe containing a [^131^I]-NaI solution with a calibrated activity concentration directly traceable to high-resolution gamma spectrometry (more details in the supplementary data).

### Gamma counting, GC

Gamma counting activity measurements were done in a Cobra II model 5003 gamma counter (Canberra-Packard, Schwadorf, Austria) using a measurement protocol optimized to limit the overall measurement uncertainty (cfr. details in the supplementary data).

The activity calibration of GC measurements was directly traceable to high-resolution gamma spectrometry (cfr. details in a Sect below). The calibration procedure of GC (cfr. details in the supplementary data) was reproducible within a 2.1% relative standard deviation (SD), corresponding to an expanded uncertainty of ± 3.4% expressed at the 95.5% confidence interval (CI) (coverage factor *k* = 3.31 for a *t*-distribution with 3 degrees of freedom (4 stock solutions)).

The combined expanded uncertainty of GC tissue activity measurements (*U*_GC_) was always within ± 3.6% (expressed at the 95.5% CI), and was determined from the square root of the summation in quadrature of the uncertainty due to calibration reproducibility and the counting statistical error of the tissue sample.

### Reference measurements for SPECT and GC activity calibrations

The reference activity concentrations of all ^131^I stock solutions used for SPECT and GC activity calibrations were determined by high-resolution gamma spectrometry analysis using a high-purity germanium detector (model GC1818-7500SL; Mirion-Canberra, Meriden, USA) calibrated for photon energy and detection efficiency. More details on the measurement procedure are provided in the supplementary data. The relative statistical uncertainty of the reference activity concentration of each of the calibration stock solutions was always within 1.6% at 95.5% CI (coverage factor *k* = 2).

### Determination of SPECT activity recovery coefficients

**Fig. 1:**
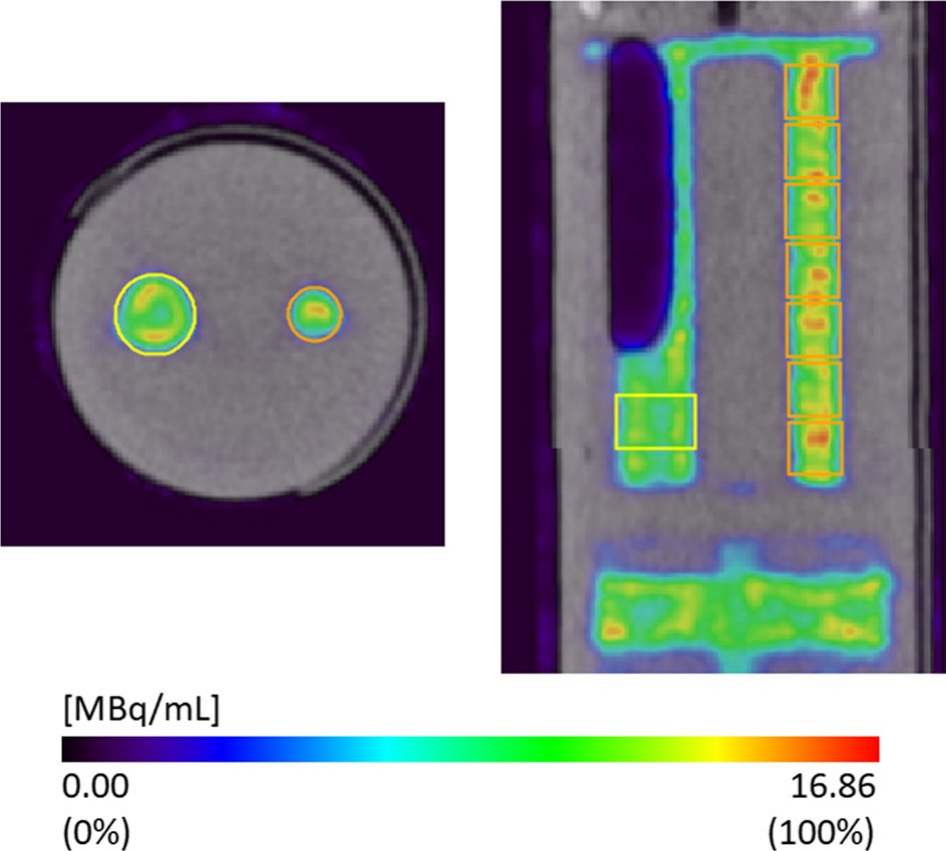
Overlays of SPECT and CT transverse (left) and longitudinal (right) section images of the physical phantom, filled with a [^131^I]-NaI solution (10.4 MBq mL^−1^). VOIs used for analysis are shown as an overlay in yellow (6 mm rod) and orange (4 mm rod) colours. A large air bubble is present inside the 6-mm-diameter rod, which limited the number of VOIs in that rod that could be used for analysis

Activity recovery coefficients (RC) were calculated based on a SPECT study of a phantom filled with a [^131^I]-NaI solution with a calibrated activity concentration of 10.4 MBq mL^−1^. The phantom (Fig. 1) is a 26-mm-diameter cylindrical container made of acrylic plastic, with two fillable compartments containing various fillable rods. Both compartments (~ 4.4 mL) were filled, but only one compartment, containing two rods of 4 and 6 mm diameter and 32 mm length, was used for analysis. A 20-min SPECT scan of the whole phantom (120 bed positions per scan, 10 s per position) was acquired. Following the SPECT scan, a CT scan of the whole phantom (55 kV X-ray tube voltage, 615 μA tube current) was acquired.

Several cylindrical VOIs were defined at the centre of each rod, with a diameter equal to the diameter of the rod and a length of 4 mm, as shown in Fig. 1. The mean activity concentration of each VOI (*AC*_*VOI*_) was evaluated. Image quantification was done with AMIDE 1.0.4 [20]. The RC was calculated, for each rod, as the ratio of the mean of the VOI activity concentrations determined in all the rod VOIs ($\overline{AC}_{VOIs}$) to the reference activity concentration of the ^131^I solution at the start of the SPECT scan (*AC*_*ref*_).

1 $$RC = \frac{{\overline{AC}_{VOIs} }}{{AC_{ref} }}$$

For the RC of the 4-mm rod, 7 VOIs were used to estimate $\overline{AC}_{VOIs}$. Because of the presence of a large air bubble in the 6-mm rod (cfr. Figure 1), only one VOI was drawn on that rod and was used to estimate the rod RC. Thus, for the RC of the 6-mm rod $\overline{AC}_{VOIs}$ is equal to *AC*_*VOI*_.

To assess the impact of lower counts on activity recovery, the phantom scan was reconstructed using only 10.0%, 5.0%, 2.5%, 1.0%, 0.5% and 0.1% of the counts from the list-mode data. These reconstructions emulate scans with lower activity concentrations equivalent, respectively, to 1.044, 0.522, 0.261, 0.104, 0.052 and 0.010 MBq mL^−1^. For each activity concentration reconstructed, the statistical uncertainty of activity recovery (*U*_Rec_) due to the regional (VOI) variability of the SPECT image counts within the 4-mm rod was estimated, at the 95.5% CI, as:

2 $$U_{{{\text{Re}} c}} = \frac{{k \cdot \sigma_{VOIs} }}{{\overline{AC}_{VOIs} \cdot \sqrt {n_{VOIs} } }}$$

where *σ*_*VOIs*_ is the standard deviation of the VOI concentrations determined in all the rod VOIs (*n*_*VOIs*_ equal to 7 VOIs); and *k* is the coverage factor, which is equal to 2.52 for a *t*-distribution with 6 (*n*_*VOIs*_ − 1) degrees of freedom. This metric captures the statistical uncertainty in the activity recovery in the VOIs, and is used in this study as an indicator of the precision of the SPECT reconstruction to recover the activity in mouse kidneys with varying levels of activity concentration.

### Animal experiments

**Fig. 2:**
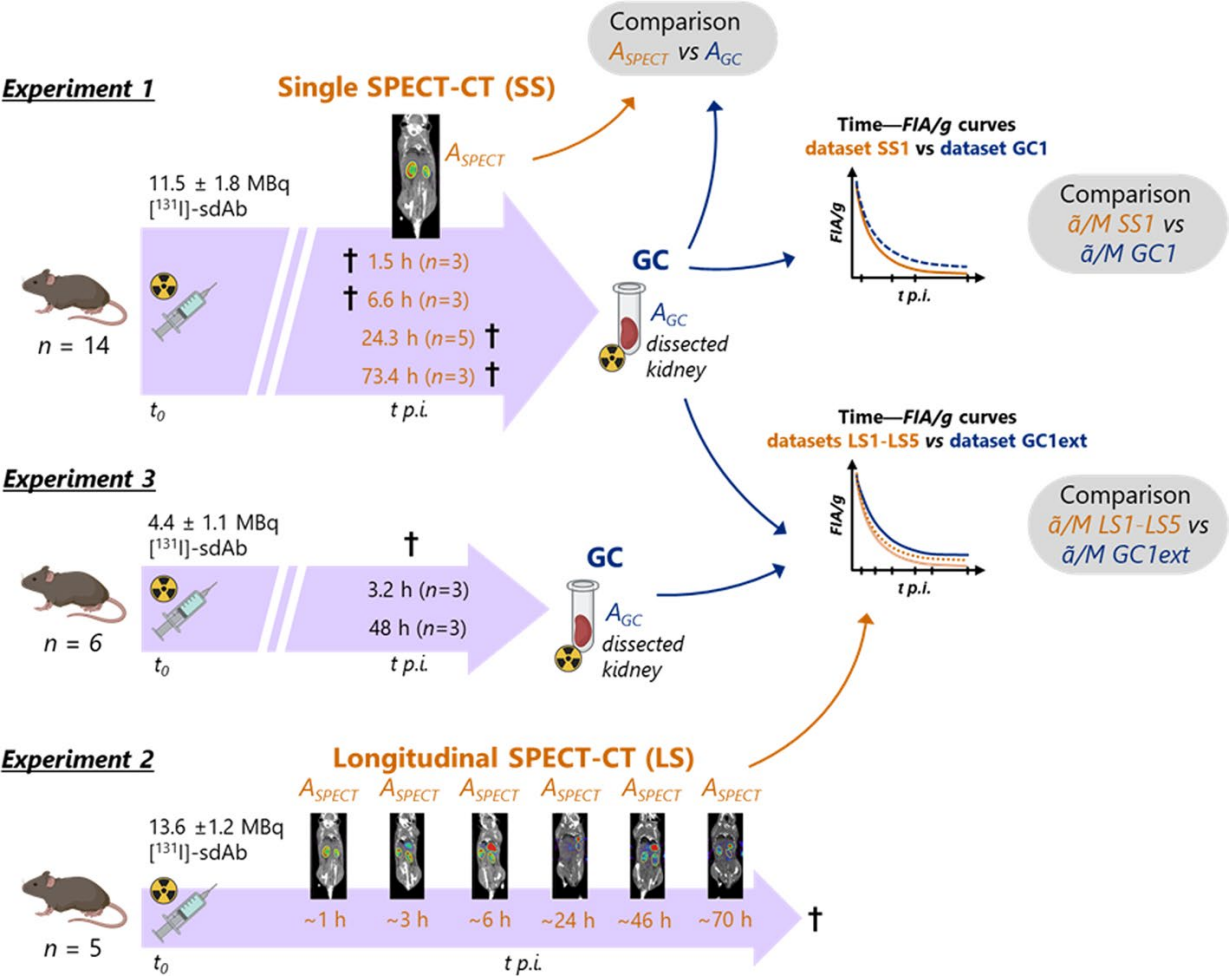
Schematic overview of the mice experiments

A schematic overview of the animal experiments is shown in Fig. 2.

The radioligand used in this study was the [^131^I]SGMIB-labelled anti-HER2 sdAb 2Rs15d, previously reported elsewhere [15]. All reagents were purchased from Sigma-Aldrich (Darmstadt, Germany) unless otherwise stated. Sodium [^131^I]iodide was purchased from Perkin-Elmer.

Anti-HER2 sdAb 2Rs15d was generated as described previously [21]. 2Rs15d sdAb was radiolabelled with ^131^I via the residualizing prosthetic group *N*-Succinimidyl 4-guanodinomethyl-3-[^131^I]iodobenzoate ([^131^I]SGMIB) and purified as reported previously [15].

All animal experiments were performed using healthy female C57BL/6 mice (8–10-week old, 19.4 ± 1.3 g body weight mean ± SD) and were conducted according to the guidelines and after approval of the Ethical Committee of the Vrije Universiteit Brussel.

#### Evaluation of accuracy of SPECT-based activity quantification (experiment 1)

An animal experiment was performed to evaluate the accuracy of SPECT-based activity quantification of ^131^I in mouse kidneys against conventional ex vivo activity measurements in a gamma counter. Mice were anesthetised by inhalation with 2% isoflurane and were intravenously injected in the tail vein with 11.5 ± 1.8 MBq [^131^I]-sdAb (5 μg sdAb). A total of 14 mice, divided into four groups (one group per time point), were imaged with SPECT/CT at around 1.5 (*n* = 3 mice), 6.6 (*n* = 3), 24.3 (*n* = 5) and 73.4 (*n* = 3) h post-injection (p.i.) of the radioligand (Fig. 2). For the first two time points (time points during fast pharmacokinetics [15]), mice were euthanized by cervical dislocation and SPECT-CT scans were done immediately on their carcasses (i.e. *post-mortem*). Both kidneys were dissected after the scans. Mice imaged at around 24 and 73 h p.i. (time points during slower pharmacokinetics [15]) were imaged in vivo, euthanized and dissected immediately after SPECT-CT imaging. A scan field of view with a 20-mm axial length located on the abdominal region comprising the kidneys was used for all the SPECT scans. Additional SPECT acquisition settings are reported in Table 1. The uptake of [^131^I]-sdAb in kidney tissues is expected to be much lower for the late time points (24 and 73 h p.i.) than for the earlier points [15]. In order to maximize the number of acquired photon counts that would be used for reconstruction, considerably longer SPECT acquisition times (within the practically and logistically feasible) were used for the late mouse scans. Following a SPECT scan, a whole-body CT scan (55 kV X-ray tube voltage, 615 μA tube current) was acquired.

**Table 1:** Acquisition settings of single SPECT scans performed in experiment 1 on 14 mice injected with the [^131^I]-labelled sdAb (dataset SS1), used for the comparison of kidney activity against gamma counting

Av. time p.i. to death [max. range of variation between mice] (h)	Animal setting	No of mice, *n*	No of bed positions per scan	Scan time per bed position (s)	Total effective scan time (min)
1.5 [± 0.0]	*Post-mortem*	3	20	66	22
6.6 [± 0.0]	*Post-mortem*	3	20	66	22
24.3^§^ [± 0.8]	In vivo	5	20	225	75
73.4* [± 2.0]	In vivo	3	20	239	80

^§^23.6 h average time p.i. to middle of scan

*72.6 h average time p.i. to middle of scan

The kidneys of all mice were weighed, and their activity was measured in the gamma counter. Radioactivity in kidney was corrected for the physical decay between the reference time (*t*_*ref*_) of the activity measurement with each technique and the mouse time of death. For GC, *t*_ref_ is the start of the GC activity measurement. For SPECT, *t*_ref_ is the start time and the middle time of the scan for *post-mortem* and in vivo scans, respectively.

The amount of ^131^I activity in the kidneys was determined from activity-calibrated SPECT images using ellipsoidal VOI. Image quantification was done with AMIDE 1.0.4 [20]. A VOI was manually drawn over each kidney based on the CT image, and its position and proportions were then fine-tuned visually according to the SPECT image. The VOI volume was adjusted to match the kidney volume (*V*) estimated from the mass of the dissected kidney (*M*) and a density of 1.04 g mL^−1^, as reported for soft tissue by Cristy and Eckerman [22].

For each mouse kidney, the percentage deviation of the activity determined with SPECT (*A*_SPECT_) from the reference activity determined with gamma counting (*A*_GC_) was calculated. The mean deviation of kidneys (both left and right) of all mice per time point was calculated. Two-tailed paired *t* tests were applied to the datasets of each time point to determine the significance of differences between SPECT and GC activity data. Statistical significance was defined as *p* < 0.05.

Additionally, for each mouse kidney, the fraction of injected activity per gram of dissected tissue (*FIA*/*g*) was calculated. For each activity quantification technique, the pharmacokinetic profile was derived from the kidney *FIA*/*g* of all mice (*n* = 14) as a function of time p.i., and the time-integrated activity coefficient (*ã*) per gram of tissue (*M*) was estimated (cfr. calculation method in a Sect. below). These time–*FIA*/*g* datasets are hereafter referred to as datasets GC1 (for gamma counting data, *n* = 14) and SS1 (for single SPECT data, also *n* = 14).

#### Pharmacokinetic assessment using longitudinal SPECT imaging (experiment 2)

A longitudinal quantitative SPECT-CT imaging study was performed on 5 mice to evaluate the feasibility of deriving mouse-specific pharmacokinetic information of the [^131^I]-sdAbs. All mice were intravenously injected with 13.6 ± 1.2 MBq [^131^I]-sdAb. Sequential SPECT-CT scans were performed in vivo on each mouse, starting at approximately 1, 3, 6, 24, 46 and 70 h p.i. of the radioligand (Fig. 2). A scan field of view with a 20-mm axial length located on the abdominal region comprising the kidneys was used for all the SPECT scans. Additional SPECT acquisition settings are reported in Table 2. Following a SPECT scan, a whole-body CT scan (55 kV X-ray tube voltage, 615 μA tube current) was acquired. Immediately after the last scan, mice were euthanized and both kidneys were dissected and weighed. Kidney activities were determined from SPECT images using the same methods as in mice experiment 1 and using as a reference time (*t*_*ref*_) the middle time of each SPECT scan. Kidney uptake (in *FIA*/*g*) was calculated at each scan time point, and the time-integrated pharmacokinetic parameter *ã*/*M* was estimated (cfr. calculation details in a Sect. below) from the pharmacokinetic profile of each mouse. These time–*FIA*/*g* datasets are hereafter referred to as datasets LS1, LS2, LS3, LS4 and LS5 (i.e. one dataset per mouse).

**Table 2:** Acquisition settings of the longitudinal SPECT scans performed in experiment 2, in vivo*,* on 5 mice injected with the [^131^I]-labelled sdAb, corresponding to datasets LS1–LS5

Av. scan start time p.i. [max. range of variation between mice] (h)	No of bed positions per scan	Scan time per bed position (s)	Total effective scan time (min)
1.0 [± 0.0]	20	51	17^§^
3.3 [± 0.0]	20	66	22
6.0 [± 0.1]	20	66	22
24.2 [± 2.0]	20	150	50
46.3 [± 3.7]	20	239 (159)*	80 (53)*
70.2 [± 3.3]	20	239 (159)*	80 (53)*

^§^Scan duration limited by the start of the scans for the next time point

*Because of a logistical limitation, this specification was used for the two mice from datasets LS1 and LS2

#### Pharmacokinetic assessment ex vivo using gamma counting (experiment 3)

In order to set a reference for comparison for the *ã*/*M* values determined from longitudinal SPECT scans (experiment 2) based on time–*FIA*/*g* data derived at similar time points, kidney uptake was assessed further with GC, this time at 3.2 and 48 h p.i. of the radioligand. No SPECT imaging was performed on these mice (*n* = 3 per time point), which allowed to inject each mouse with a lower amount of activity of [^131^I]-sdAb (4.4 ± 0.1 MBq) than in the other (imaging) experiments. The time-integrated pharmacokinetic parameter *ã*/*M* was estimated (cfr. calculation method in the following Sect.) from all the kidney *FIA*/*g* determined with GC in this experiment (3.2 and 48 h p.i.) and in experiment 1 (1.5, 6.6, 24.3 and 73.4 h p.i). This time–*FIA*/*g* dataset is hereafter referred to as dataset GC1ext (*n* = 20).

#### Pharmacokinetic modelling

Kidney pharmacokinetic data were analysed by nonlinear least squares fitting (MATLAB R2019a, MathWorks, Massachusetts, USA) to a mathematical function of time elapsed p.i. (*t*). A negative power function with two coefficients (*c*_*1*_ and *c*_*2*_) (Eq. 3) was chosen among the various mathematical functions considered (cfr. supplementary data). Eight datasets of *FIA*/*g* as a function of time (datasets GC1, SS1, GC1ext and LS1–LS5) were analysed. The Pearson’s correlation coefficient (*R*^2^) was used to quantify goodness of fit.

3 $$FIA\left( t \right)/g \cong c_{1} t^{{ - c_{2} }}$$

Two values of the time-integrated pharmacokinetic parameter *ã*/*M* of the left kidneys were calculated from integration over two periods of time. A first value (*ã*_*t0→∞*_/*M*) was estimated from mathematical integration from injection (*t* = 0) to infinity in two parts. Kidney uptake was assumed to be zero at *t* = 0 and to increase linearly over time until a peak value at *t* = 1.1 h (earliest first measured time point of all datasets), which was calculated from the power function fit. Then, from *t* = 1.1 h until infinity, kidney uptake was assumed to follow the power function fit. A second value (*ã*_*t1→∞*_/*M*) was calculated from mathematical integration of the power functions only from 1.5 h to infinity. The purpose of estimating *ã*_*t1→∞*_/*M* was to investigate the impact of the assumption (extrapolation) of time–*FIA*/*g* data between injection and the first time point p.i. covered by most datasets (1.5 h) on the comparison of *ã*_*t0→∞*_/*M* values of SPECT and GC.

Using the time-dependent fit function derived from pharmacokinetic dataset GC1, the kidney *FIA*/*g* was estimated at the time point halfway between the start and the end of in vivo SPECT scans of dataset SS1, and at the mouse time of death, the deviation between the resulting *FIA*/*g* values was calculated. This was used to estimate the potential bias (over-response) associated with SPECT measurements performed in vivo when compared with GC measurements, due to the estimated (expected) decrease in radioligand uptake in the kidneys during the (scan) time prior to death.

## Results

### SPECT activity recovery coefficients and the effect of activity concentration

The RC determined from the phantom image with 10.4 MBq mL^−1^ was 0.78 and 0.85, respectively, for the 4- and 6-mm-diameter rods, indicating a partial (respectively, 78% and 85%) recovery of the reference activity present within the rods.

**Fig. 3:**
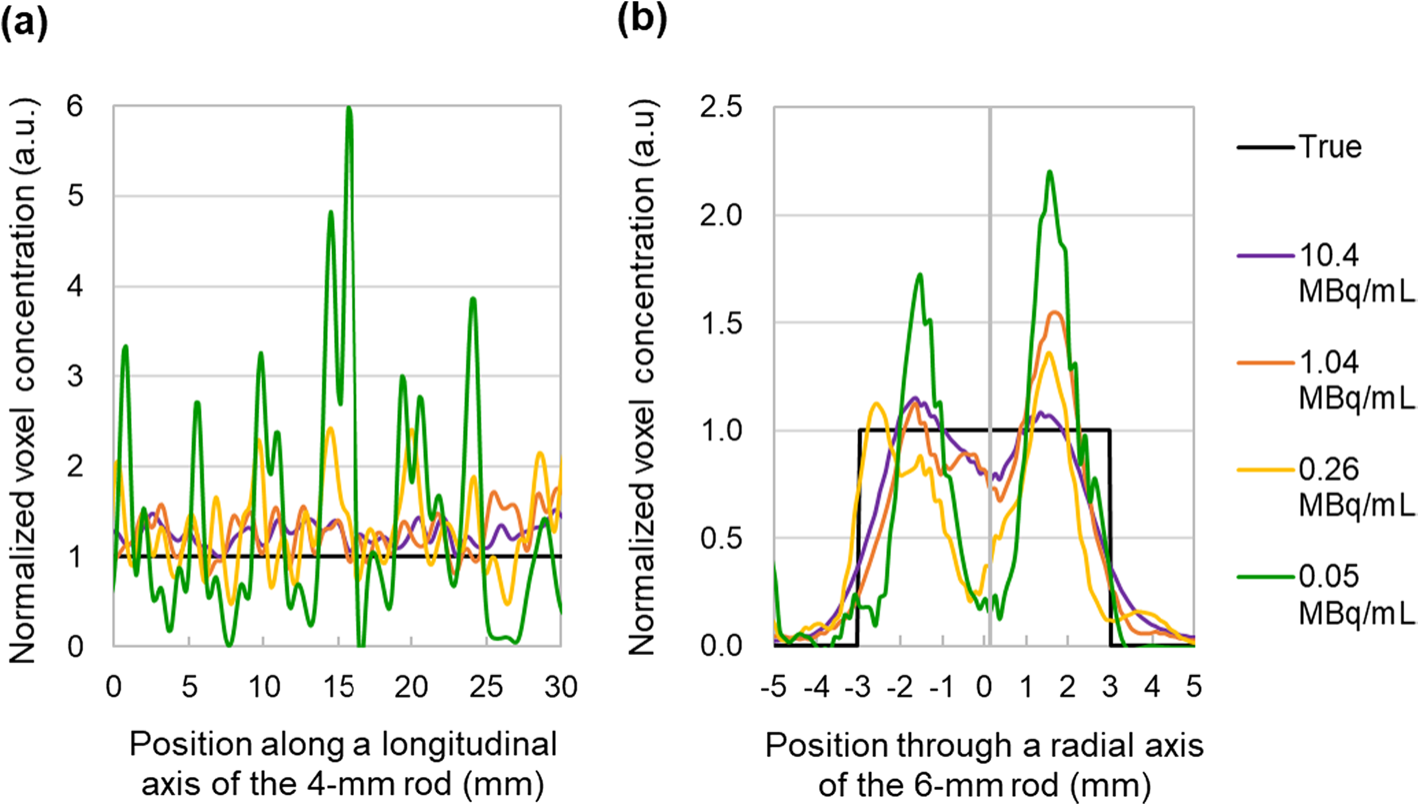
Longitudinal and radial profiles through, respectively, the 4-mm (**a**) and the 6-mm (**b**) rods, for various (emulated) ^131^I activity concentrations (same series legend for the two sub-figures) of the phantom scan

**Fig. 4:**
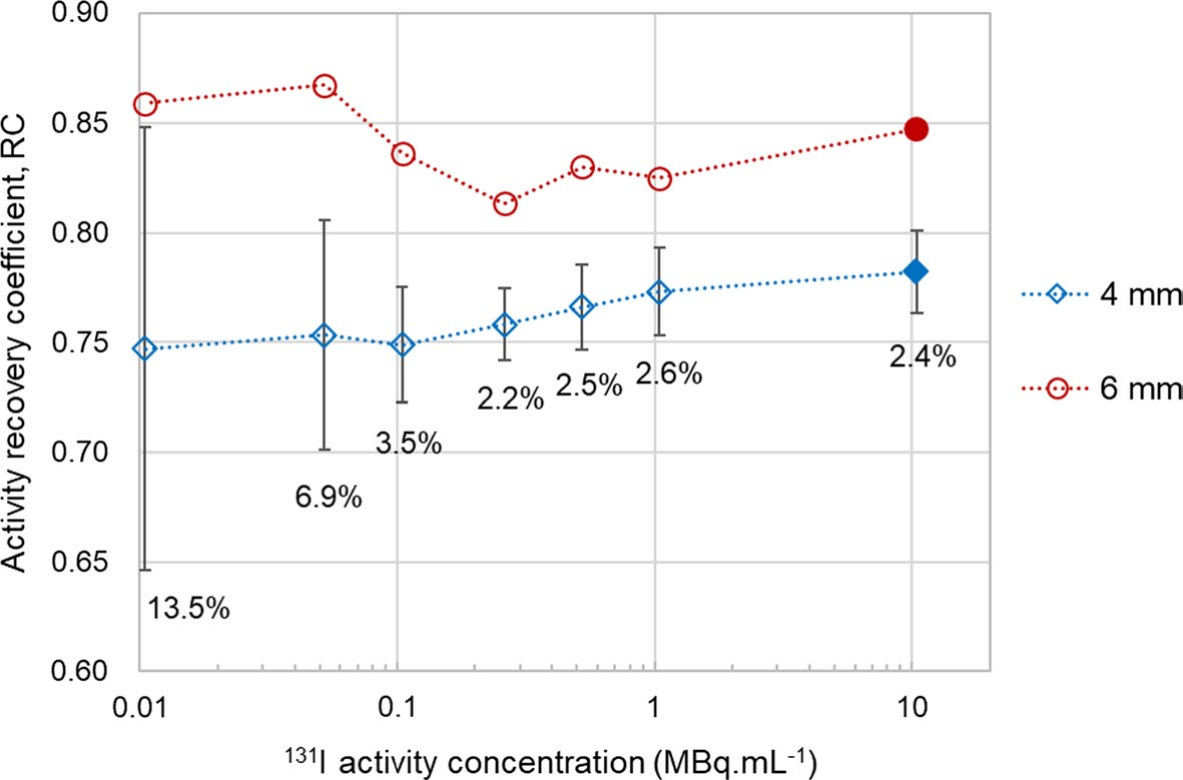
RC for the 4-mm and the 6-mm rods for various ^131^I activity concentrations of the phantom scan. Empty symbols indicate RC values obtained from reconstructions emulating activity concentrations lower than the activity concentration actually imaged (10.4 MBq mL^−1^, RC values indicated by filled symbols). Error bars and labels (shown only for the 4-mm rod) indicate ± the expanded statistical uncertainty of activity recovery, *U*_Rec_, at the 95.5% CI

The effect of lower activity concentrations (from reconstructing with fewer counts) in the local (voxel) and regional (VOI) activity recovery is shown in Figs. 3 and 4, respectively. Inside the 6-mm rod (Fig. 3b), an overshoot appears somewhat near the rod edge, whereas an undershoot appears in the centre, which we tentatively attribute to the Gibbs effect. The overshoot–undershoot delta becomes more pronounced in the reconstructions of lower activity concentrations. For both rods, the variability of the locally reconstructed activity concentration within the rod increases with decrease in activity concentrations. At a regional level, the RC of the 4-mm rod (mean of 7 VOIs) decreases by just a few per cent for the lowest concentrations, yet the statistical uncertainty in VOI activity recovery, *U*_Rec_, significantly increases for concentrations below 0.1 MBq mL^−1^. Such statistical uncertainty is implicit in the RC values of the 6-mm rod, because they were estimated from just one VOI. This is likely the reason why the RC of the 6-mm rod shows a less consistent variation with decreasing activity concentrations, compared with the RC of the 4-mm rod.

### Mice studies

**Fig. 5:**
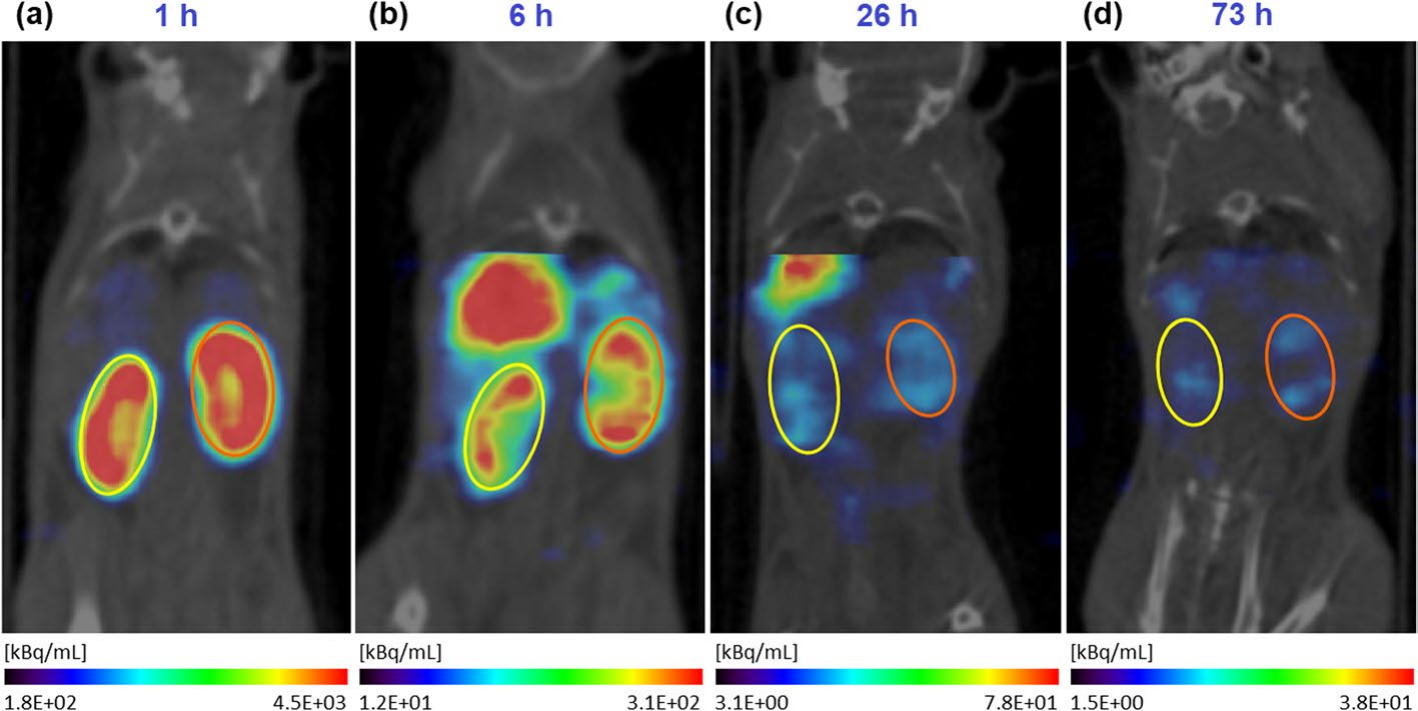
Overlays of SPECT and CT section images through the kidneys, sequentially acquired in vivo on the mouse of dataset LS5, at around 1 (**a**), 6 (**b**), 26 (**c**) and 73 (**d**) h p.i. of the [^131^I]-sdAb. VOIs used for activity quantification are shown in each sub-figure as an overlay in yellow (left kidney) and orange (right kidney) colours. High uptake above left kidney of sub-figures (**b**) and (**c**) corresponds to liver tissue. To enhance the visibility of kidney tissues, the activity concentration data of each sub-figure is scaled to 2% (minimum value) and 50% (maximum value) of the maximum voxel value of the SPECT image

Representative examples of the SPECT and CT images and the VOIs used for quantification are shown in Fig. 5. At early time points (≤ 6 h), the predominant uptake in kidneys is clearly visualized on the SPECT images. At late time points (≥ 24 h), the SPECT images appear less uniform, the radioligand is more widespread in other abdominal tissues (e.g. liver, intestines), and kidney uptake is less evident.

#### Accuracy of SPECT-based activity quantification

**Fig. 6:**
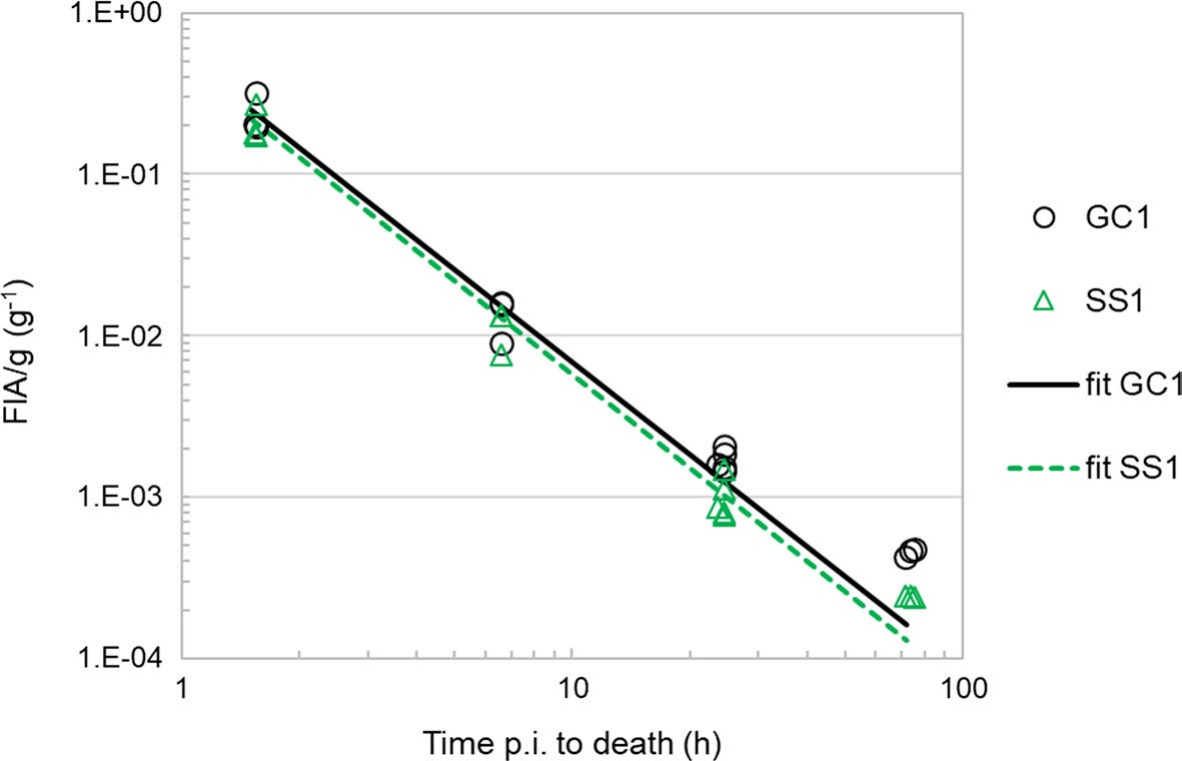
Time–*FIA*/*g* data of the left kidney determined with SPECT (dataset SS1) and with GC (dataset GC1), of 14 mice injected with the [^131^I]-labelled sdAb. The curves indicate the power function fit of each dataset (cfr. function coefficients in Table 4)

**Fig. 7:**
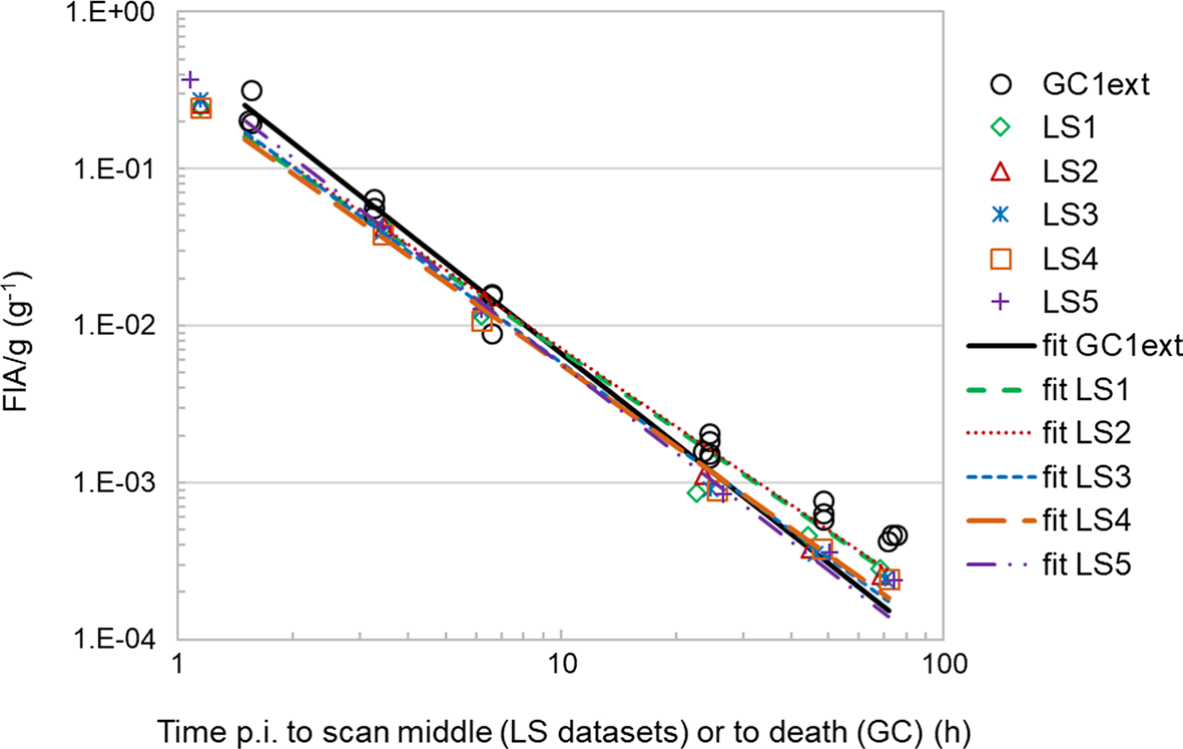
Time–*FIA*/*g* data of the left kidney determined with longitudinal in vivo SPECT imaging (datasets LS1–LS5) and with GC (dataset GC1ext), of, respectively, 5 (SPECT) and 20 (GC) mice injected with the [^131^I]-labelled sdAb. The curves indicate the power function fit of each dataset (cfr. function coefficients in Table 4)

Figure 6 illustrates the pharmacokinetic profile of the [^131^I]-sdAb in the kidney, for the datasets used to evaluate the accuracy of SPECT-based kidney activity quantification (SS1 *vs* GC1). Figure 7 illustrates the mouse-specific pharmacokinetic profile of the [^131^I]-sdAb for each of the *FIA*/*g* datasets acquired with longitudinal in vivo SPECT in a single mouse (LS1–LS5), as well as the pharmacokinetic profile of the GC dataset with additional time points (GC1ext).

The mean kidney *FIA*/*g* (of left and right kidneys of all mice per time point) determined via GC are listed in Table 3. The radioligand uptake in the kidneys is the highest at the earliest time point and decreases rapidly over time, with only about 5%, 0.6% and 0.2% of the activity measured at 1.5 h remaining in the kidney after, respectively, 6.6, 24 and 73 h p.i.

**Table 3:** Overview of the mean values (of left and right kidneys of all mice per time point) of the *FIA*/*g* determined with GC, the reference activity concentration *A*_*GC*_/*V* and the percentage deviation of *A*_SPECT_ from *A*_GC_. Also, the mean values (of all mice per time point) of the total number of scan counts acquired in the photo-peak window and the percentage of these counts that were used as background and scatter are indicated, for the SPECT acquisitions of dataset SS1

Av. time p.i. to death (h)	No of mice, *n*	GC (dataset GC1)		Av. % dev. *A*_*SPECT*_ from *A*_*GC*_ (%)	SPECT (SS1 dataset)	
		Av. *FIA*/*g* [± SD] (g^−1^)	Av. *A*_*GC*_/*V* (kBq mL^−1^)		Av. of total scan counts acquired in photo-peak window	Av. of % of photo-peak counts used as background (%)
1.5	3	2.6E−01 [± 6.5E−02]	2.89E+03	− 12	4.46E+06	11
6.6	3	1.4E−02 [± 3.2E−03]	1.49E+02	− 13	8.05E+05	22
24.3	5	1.7E−03 [± 2.2E−04]	1.76E+01	− 40	1.03E+06	44
73.4	3	4.5E−04 [± 1.8E−05]	5.21E+00	− 46	8.56E+05	51

**Fig. 8:**
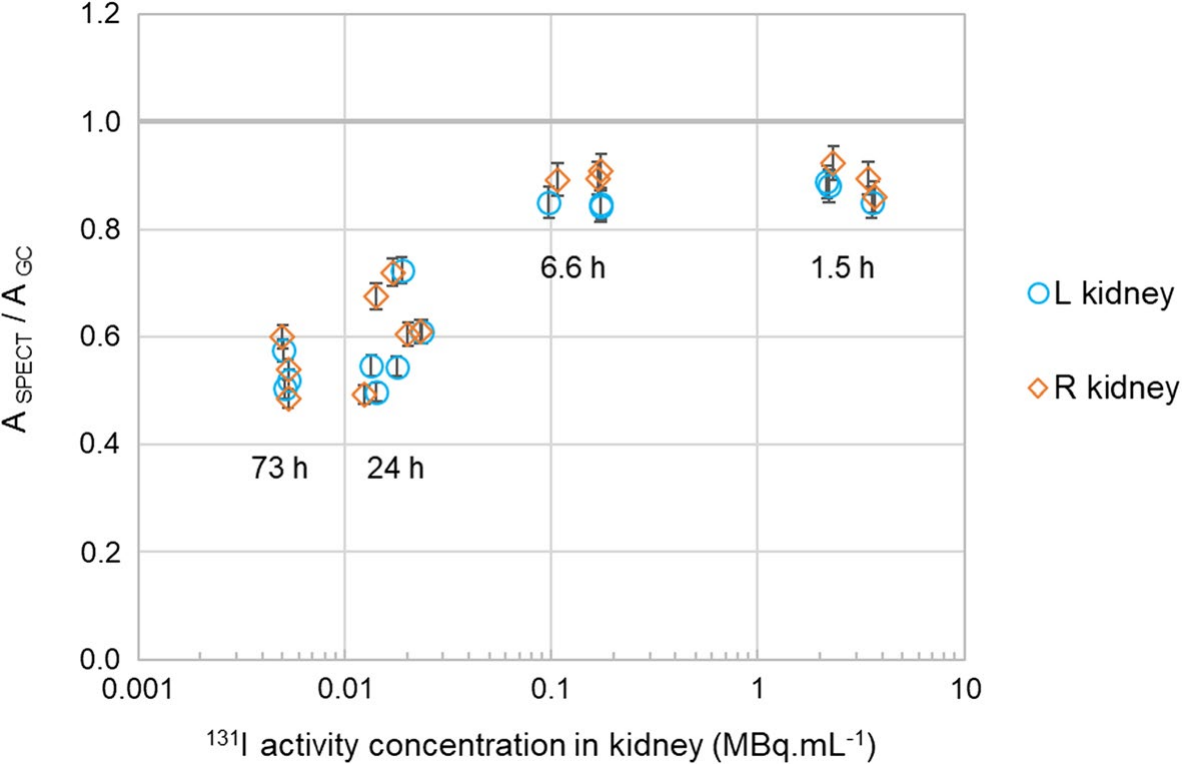
Ratios between the ^131^I activity in the kidney determined with SPECT (*A*_SPECT_) and the reference activity determined with GC (*A*_GC_) as a function of the reference activity concentration in the kidney (*A*_*GC*_/*V*), for all mice (*n* = 14) of datasets GC1 and SS1. Each data symbol corresponds to one kidney of one mouse. Labels indicate the approximate time point of the pharmacokinetic profile to which a group of data symbols belong. Error bars indicate ± the expanded combined uncertainty of only GC measurements, *U*_GC_, at the 95.5% CI

SPECT-based activities tend to underestimate the reference activity by an amount that is related to the ^131^I activity concentration in the kidney, which is dependent on the time point of the pharmacokinetic profile of the [^131^I]-sdAb (see Table 3; Fig. 8). This underestimation is statistically significant for all time points (*p* value < 0.01).

#### Time-integrated pharmacokinetic parameter ã/M

The coefficients of the power function fits and the time-integrated activity coefficient per mass of tissue *ã*/*M* of all GC and SPECT measurement datasets are listed in Table 4. The *R*^2^ values of the fit functions were 0.936, 0.925 and 0.929, respectively, for datasets SS1, GC1 and GC1ext, indicating a strong correlation between the measured *FIA*/*g* values and the values estimated by the power function fit for the three datasets, derived from multiple sacrificed mice. The *R*^2^ values of the fit functions of the LS datasets were all > 0.999, indicating an even stronger correlation between measured and curve fit-estimated *FIA*/*g* data.

**Table 4:** Overview of power function fit coefficients (*c*_1_ and *c*_2_) and time-integrated activity coefficient per mass of tissue (*ã*/*M*) obtained from various datasets of the left kidney *FIA*/*g* determined with GC or SPECT; and the effect of applying a correction for partial recovery to SPECT activities

Dataset [technique]	No of mice, *n*	No of time points	Time integration from injection until infinity						Time integration from 1.5 h until infinity			
			Without RC-based correction				With RC-based correction = 1/0.85		Without RC-based correction		With RC-based correction = 1/0.85	
			Function coefficients		*ã*_*t0→∞*_/*M* (h g^−1^)	% dev. from GC1ext *ã*_*t0→∞*_/*M* (%)	*ã*_*t0→∞*_/*M* (h g^−1^)	% dev. from GC1ext *ã*_*t0→∞*_/*M* (%)	*ã*_*t1→∞*_/*M* (h g^−1^)	% dev. from GC1ext *ã*_*t1→∞*_/*M* (%)	*ã*_*t1→∞*_/*M* (h g^−1^)	% dev. from GC1ext *ã*_*t1→∞*_/*M* (%)
			***c***_***1***_** (g**^−**1**^**)**	***c***_***2***_								
GC1 [GC]	14	4	0.547	1.90	0.809	1	–	–	0.422	2	–	–
SS1 [SPECT]	14	4	0.481	1.92	0.696	− 13	0.819	2	0.357	− 14	0.421	2
GC1ext [GC]	20	6	0.551	1.92	0.803	–	–	–	0.414	–	–	–
LS1 [SPECT]	1	6	0.314	1.65	0.597	− 26	0.703	− 12	0.367	− 11	0.432	4
LS2 [SPECT]	1	6	0.326	1.66	0.619	− 23	0.728	− 9	0.380	− 8	0.447	8
LS3 [SPECT]	1	6	0.348	1.77	0.579	− 28	0.681	− 15	0.328	− 21	0.386	− 7
LS4 [SPECT]	1	6	0.314	1.74	0.542	− 32	0.639	− 20	0.315	− 24	0.371	− 10
LS5 [SPECT]	1	6	0.428	1.88	0.645	− 20	0.759	− 5	0.342	− 17	0.402	− 3

Concerning the comparison of SPECT against GC (Table 4), the *ã*_*t0→∞*_/*M* value of SPECT dataset SS1 was − 14% lower than that of GC dataset GC1 determined on the same mice. Larger deviations from the reference *ã*_*t0→∞*_/*M* value of dataset GC1ext are observed with longitudinal SPECT datasets (in vivo scans), ranging from − 20 to − 32% (− 26% mean deviation of 5 mice), depending on the mouse investigated (Table 4). Yet, for *ã*_*t1→∞*_/*M*, the range of deviation from the reference CG1ext value is within − 24% for all LS datasets (− 16% mean deviation of 5 mice). Thus, there is a better agreement between the time-integrated parameters *ã*/*M* of the longitudinal SPECT scans and the reference GC dataset GC1ext when the extrapolation of time–*FIA*/*g* data required for the period of time between injection and *t* = 1.5 h is omitted when calculating *ã*/*M*.

The mean semi-axes (± SD) of the kidney ellipsoidal VOIs of all the mice imaged with SPECT were 2.3 (± 0.14), 2.9 (± 0.16) and 5.1 (± 0.23) mm. Thus, for a PVE analysis one may approximate a mouse kidney roughly as a rod with a radius of 3 mm. The RC of a kidney when the activity concentration in adjacent tissues is negligible would then be around 0.85, corresponding to the RC of the 6-mm-diameter rod (for an activity concentration of 10.4 MBq mL^−1^). Aiming to compensate to some extent for partial recovery of SPECT images in the pharmacokinetic assessment, a correction factor equal to the inverse of the RC of the 6-mm rod (*i.e.* 1/0.85 equal to 1.176) was applied to all kidney activities determined with SPECT. New curve fits were determined and both *ã*/*M* values (*ã*_*t0→∞*_/*M* and *ã*_*t1→∞*_/*M*) were estimated for the SS1 and LS datasets (Table 4). This correction increases all the *ã*/*M* values by a factor equal to the inverse of the RC. For *ã*_*t0→∞*_/*M*, the RC-based correction reduces the range of deviation from the reference GC1ext value to just 2% for dataset SS1 and to a value within − 20% for all the LS datasets (Table 4). When considering a partial period of time for time integration (*ã*_*t1→∞*_/*M* values), the range of deviation from the GC1ext value is reduced to a value within ± 10% for all LS datasets.

## Discussion

This study investigated the accuracy of activity quantification in mouse kidneys after injecting a radioligand with significant kidney uptake, using a commercial micro-SPECT/CT system.

Ex vivo gamma counting was used as the reference method for determining the reference ^131^I activity in mouse kidneys because of its high measurement sensitivity and quantitative accuracy and its widespread use in preclinical research. The measurement protocol was optimized to limit (or control) some systematic (activity calibration, linearity, crosstalk, volume effects) and statistical (measurement counts) uncertainty of GC measurements to increase the confidence in the reference values.

To limit the influence of errors associated with reproducibility and accuracy of the activity calibration procedures, activity measurements with GC and SPECT were directly traceable to high-resolution gamma spectrometry.

### SPECT phantom study

Significant PVE are expected for the imaging tasks investigated because of the rather large pinhole diameter (1.6 mm) of the SPECT collimator used, which enhances photon detection sensitivity at the expense of degrading the system spatial resolution [2, 4]. Additionally, imaging of ^131^I can be challenging because of the higher energy of the photons used for imaging (365 keV) and the presence of significant photon emissions of even higher energies (around 9% yield of 637 and 722 keV gammas). The latter increases pinhole edge and collimator penetration effects, both of which have a degrading effect on spatial resolution and therefore on PVE [2].

Generally, for the phantom setting, PVE lead to an underestimation of the activity quantified with rod-delimited VOIs. As expected, the influence of PVE is larger for smaller objects; therefore, the RC is lower for the 4-mm rod (0.78) compared with the 6-mm rod (0.85).

Both Gibbs and noise artefacts are known image-degrading effects related to image reconstruction methods in SPECT [7]. Gibbs artefacts can lead to a positive bias (overestimation), or under certain circumstances a negative bias (underestimation), in activity quantification [23, 24].

For the imaging settings of the phantom scan, the reconstruction of lower activity concentrations impacts mostly the precision (*U*_Rec_) rather than the bias of activity quantification with the regional VOI. Low-counts reconstructions are more prone to noise, which can increase the statistical uncertainty in activity quantification in a VOI, as indicated by the larger *U*_Rec_. The somewhat lower RC values of the 4-mm rod observed with decreasing concentration of counts might be due to an additional negative bias from the reconstruction, and/or due to the need of more iterations. The latter is a relevant aspect since the image reconstruction of regions with lower counts are known to converge more slowly than high-count regions [25].

To note, the count fractionation used in this study to emulate lower activity (or shorter) scans does not discriminate between counts associated to ^131^I radiations and true background counts. Background signal is independent on the activity scanned and its count rate should remain constant over any acquisition time. The emulated low-count image has a fixed signal-to-background ratio and is less affected by fluctuations in the background. This means that for actual low activity-concentration studies the decrease in the RC and the increase in *U*_Rec_ might possibly be larger (i.e. worse) than predicted here from emulated low-count images (Fig. 4).

### Accuracy of SPECT-based activity quantification in mouse kidneys

With the imaging and quantification settings used in this study, micro-SPECT tends to underestimate the ^131^I activity of [^131^I]-sdAb present in mouse kidneys. The amount of underestimation is considerably larger for the late time points of the pharmacokinetic profile of the [^131^I]-sdAb (≥ 24 h p.i.), when the kidney activity concentrations are below 26 kBq mL^−1^.

To limit the reproducibility uncertainty in SPECT quantification due to an arbitrary selection of VOI size, a consistent approach for activity quantification was used in which for each mouse kidney the size of the VOI was adjusted to match the estimated volume of the kidney. Ellipsoidal VOIs were used, generally resembling well the shape of mouse kidneys, while providing a convenient and reproducible solution for VOI delineation of SPECT images. Although an ellipsoidal VOI does not provide an optimal fit of the shape of just any mouse kidney, the error (variability) in activity quantification resulting from this approach is expected to be smaller than the error from using a subjective VOI size. Yet, the use of ellipsoidal VOIs may pose an uncertainty in the activity quantification with SPECT, because the VOI might not fully enclose all kidney tissue and/or might also enclose surrounding tissue. This could lead to an overestimation or an underestimation of the activity in the kidney, depending on the ratio of activity between kidney and surrounding tissues. An analysis on this source of uncertainty was not possible in this study, since the visibility of kidney tissues in the CT images was insufficient to enable the use of detailed CT-based VOIs.

The RC of the 6-mm rod (0.85) can be used to approximate the amount of partial recovery in the kidneys of this study, for when the activity concentration in adjacent tissues is negligible. This could explain the *A*_SPECT_/*A*_GC_ values obtained at 1.5 and 6.6 h and to some extent at 24 and 73 h p.i. (cfr. Figure 8). Yet, PVE are dependent not only on the size, but also the shape of the tissue/object of interest and the activity concentration ratio between the tissue/object and surrounding regions [4, 26]. The phantom consists of hot rods filled with uniform activity concentration and embedded in a cold medium. Kidneys have an approximately ellipsoidal shape, and the activity biodistribution in the kidney and surrounding tissues is not uniform and changes over time. Thus, PVE might not be exactly the same for the phantom setting and the activity distribution settings of [^131^I]-sdAb in mouse kidneys.

For the lower *A*_SPECT_/*A*_GC_ values obtained at 24 and 73 h, we hypothesize that this bias is related to the (larger) impact of low-counts image reconstruction effects. The amount of counts potentially useful for imaging the uptake of [^131^I]-sdAb in the kidney are proportional to several of factors. One of them is the amount of ^131^I activity present in the kidney at the moment of the scan, which depends on the radioligand pharmacokinetics and the activity administered to the mouse. Another factor is the total time spent by the scan in each bed position. When all the activity is located within the scan FOV, the counts useful for imaging of the FOV may be approximated as the total counts acquired in the photo-peak window minus the amount of these counts estimated as background and scatter [9]. Overall, the pharmacokinetics of the [^131^I]-sdAb in mouse healthy tissues are fast, with less than 3% of the injected activity remaining in the healthy mouse body after 24 h of administration. Although for the late scans at 24 and 73 h p.i. about 3.5-times longer acquisition durations are used (cfr. Table 1), the counts potentially useful for imaging the uptake in tissues within the FOV in these scans are still in the order of 50 and 150 times lower compared to the earliest scans (cfr. Table 3). The amount of counts useful for imaging specifically the uptake of [^131^I]-sdAb in the kidneys would be even lower, since at late time points the activity is more widespread among other tissues located within the scan FOV (e.g. liver, intestines). Another relevant aspect is the background-and-scatter to photo-peak counts fraction (BgSc/PP). The BgSc/PP fraction was significantly larger for the late scans (around 44% and 51%, respectively, at 24 and 73 h, cfr. Table 3) compared to the earlier scans (11% and 22%, respectively, at 1.5 and 6.6 h, cfr. Table 3), which renders the reconstructions of the late scans more prone to the effects of noise. The analysis of the RC of the 4-mm rod indicated a larger bias and statistical uncertainty in activity recovery when reconstructing with lower counts. The number of photo-peak counts acquired and the BgSc/PP fraction are similar for the phantom scan (approximately 3.7E+06 counts for the equivalent of 20 bed positions and 13% BgSc/PP) and the mouse scans at 1.5 h p.i. (around 4.5E+06 photo-peak counts per scan and 11% BgSc/PP). Thus, the magnitude of the effects observed in the RC of the 4-mm rod may be considered representative of the earliest scans. These effects might be worse for the late scans, because of their larger BgSc/PP fractions.

Finally, regarding the influence of the variation of kidney uptake over (scan) time in the evaluation of accuracy of SPECT activities, the kidney activities at the moment of the late scans would still be very low despite the 2-to-5%-higher uptake expected at the middle time of the in vivo scans (compared with the uptake at time of death). Therefore, the potential over-response in SPECT-based activities expected from this effect is outweighed by the effects associated to low-counts SPECT studies mentioned above.

### Time-integrated pharmacokinetic parameter *ã*/*M*

For a fixed mathematical model, the accuracy of the estimation of the time-integrated *ã*/*M* coefficient depends on the accuracy of the time–*FIA*/*g* data, the goodness of fit of the mathematical model to the data and the predictive power of the model outside of the data sample.

The power function fits of SPECT dataset SS1 and GC datasets GC1 had similar *R*^2^ values and were based on the same set of sampled time points. Thus, differences in the *ã*/*M* values estimated from these time–*FIA*/*g* datasets are most likely due to differences in the kidney activity values measured with the two techniques. Because of the much larger amount of activity present in the kidneys at early time points and the fast pharmacokinetics of the [^131^I]-sdAb, the *ã*/*M* estimation is influenced mostly by the activity at the early time points. Despite the large (≥ 28%) underestimations associated with *A*_*SPECT*_ values of late time points, the *ã*/*M* values (either *ã*_*t0→∞*_/*M* or *ã*_*t1→∞*_/*M*) are only around 15% lower with SPECT SS1 compared with GC and as such follow approximately the level of underestimation of *A*_*SPECT*_ of early time points (~ 12%). This is also the reason why a recovery correction of just 1/0.85 for all *A*_SPECT_ values was sufficient to reduce the difference between the *ã*/*M* values estimated with GC and SPECT SS1 to just 2% (cfr. Table 4).

It should be noted that the RC based on the phantom setting might be useful to compensate for partial recovery but it is clearly limited by several parameters affecting PVE and activity recovery. Based on the analyses of this study, the RC of the 4-mm rod bears a statistical uncertainty of about 2.4% (*U*_*Rec*_). It is reasonable to assume that the RC of the 6-mm rod would bear a similar statistical uncertainty. Additionally, the compensation based on that RC might bear a bias of few per cent due to differences in the activity concentration of the solution used to fill the phantom and the concentration of activity in mouse kidneys at different time points. The number of potentially useful counts acquired per bed position and the BgSc/PP fraction were not the same for the phantom scan and some of the mouse scans, which affects the transferability of RC data to the mouse scans. Moreover, differences in the shape and uniformity of the activity distribution of the phantom and the mouse scans are also sources of uncertainty. The impact of these factors in the accuracy of the compensation for activity recovery was not evaluated in this study. Preclinical quantitative micro-SPECT studies would benefit from activity recovery investigations using phantoms with improved anatomical realism, such as spherical and multi-compartment objects [14, 26]. The accuracy of the compensation for partial activity recovery might be improved further by determining a curve of RC as a function of object size (e.g. rod diameter, sphere volume). However, the variation in size of the mouse kidneys used was very low (relative SD of each radius of the kidney ellipsoidal VOIs was ≤ 6%), which is expected to result in an also low variation in RC. For this reason, we assumed that the use of a diameter–RC curve would potentially not be necessary for the comparisons made in this work.

This study demonstrates the feasibility of deriving quantitative mouse-specific kidney pharmacokinetic data from longitudinal in vivo SPECT studies of kidney ^131^I activity quantification. Compared with the pharmacokinetic datasets derived from multiple mice (GC1, GC1ext, SS1), the *R*^2^ values of the fit functions were especially good (> 0.999) for the datasets derived with in vivo SPECT. The better *R*^*2*^ values might result from the fact that in a longitudinal SPECT study each mouse acts as its own control for time sampling, therefore eliminating the impact of mouse inter-variability in the pharmacokinetics assessment. This can be an important gain of SPECT over conventional GC methods when a more accurate investigation of the pharmacokinetics is of interest, such as for tissue dosimetry in targeted radionuclide therapy. The variation in the *ã*_*t0→∞*_/*M* values of different mice (LS1–LS5) and their deviations from the reference *ã*/*M* value (dataset GC1ext) might just reflect mouse inter-variability, or a combination of the latter with an error in SPECT activity quantification due to a non-negligible change in kidney uptake during the rather long SPECT acquisition especially at early time points (1.0 and 3.3 h p.i.). Shorter scan acquisitions (e.g. of few minutes) are feasible and can be used to reduce that source of uncertainty. Also the assumptions (extrapolations) made to model time–*FIA*/*g* data between injection and the earliest time point sampled contributes to the deviations of the longitudinal SPECT datasets from GC1ext. While for the LS datasets the earliest time point is at around 1.1 h p.i. (middle time of SPECT scans), for the GC datasets it is 1.5 h. This implies an additional extrapolation of time–*FIA*/*g* data in the estimation of the *ã*/*M* of GC datasets, compared with LS datasets. Although the difference in time may seem small, it is relevant for the *ã*/*M* estimation because of the fast early pharmacokinetics of the [^131^I]-sdAb [15]. Values of *ã*_*t1→∞*_/*M* are less sensitive to the model assumptions and as such are more relevant for the comparison of in vivo SPECT against GC. A more detailed analysis of the influence of the mathematical models and assumptions used for time integration on the *ã*/*M* calculation was beyond the scope of this study. This remains, however, a relevant aspect to be considered in dosimetry investigations, where estimation of *ã*_*t0→∞*_/*M* is required.

Lastly, mouse-specific absorbed dose estimates enabled by in vivo quantitative SPECT permit to evaluate dose and response on the same animal, which allows to limit the blurring effect that mouse inter-variability poses to the correlation of biological response with absorbed dose. Ultimately, this might translate into a gain in effectiveness in the task of finding and establishing sound preclinical absorbed dose–biological response relationships in targeted radionuclide therapy. Also, since in vivo SPECT obviates the need of multiple experimental groups for time sampling to assess the pharmacokinetic profile, it may allow a reduction in the overall animal burden of preclinical pharmacokinetic and biological effect investigations. This implies an important gain from an animal ethics perspective, as laid out by the 3R principle (Replacement, Reduction, Refinement) [27].

## Conclusion

Although quantification in mouse tissues using micro-SPECT can be affected by partial recovery of the imaged activity and variability in the reconstructed counts, a robust activity quantification methodology allows to derive reasonably accurate time-integrated pharmacokinetic information of the ^131^I-labelled sdAb investigated in mouse kidneys. This outcome is satisfactory, considering the benefits associated with non-toxic in vivo methods vis-à-vis analogues ex vivo methods. Overall, these gains continue to bring forward the use of micro-SPECT imaging for preclinical quantitative imaging studies of drug pharmacokinetics in basic and (back) translational research.

## Supplementary Information

**Additional file 1.** Supplementary data.

## Data Availability

The data that support the findings of this study are available from the corresponding author upon reasonable request.

## Abbreviations

[^131^I]SGMIB: *N*-Succinimidyl 4-guanodinomethyl-3-[^131^I]iodobenzoate
BgSc/PP: Background-and-scatter to photo-peak counts
CI: Confidence interval
Cps: Counts per second
CT: Computed tomography
FIA/g: Fraction of injected activity per gram of tissue
GC: Gamma counting
GC1ext: Extended gamma counting (dataset)
LS: Longitudinal single-photon emission computed tomography (dataset)
OSEM: Ordered subset expectation maximization
p.i.: Post-injection
PVE: Partial volume effects
RC: (activity) recovery coefficient
SD: Standard deviation
SS: single single-photon emission computed tomography tomography (dataset)
sdAb: Single-domain antibody fragment
SPECT: Single-photon emission computed tomography
VOI: Volume of interest
