# Supplementary material for: Assessment of mouse-specific pharmacokinetics in kidneys based on 131I activity measurements using micro-SPECT
Source: EJNMMI Phys. 2022 Feb 23;9:13. doi: 10.1186/s40658-022-00443-5 (PMC8866625; doi:10.1186/s40658-022-00443-5)
Supplement: Supplementary file 1 — Additional file 1. Supplementary data. [file 40658_2022_443_MOESM1_ESM.docx]

**Supplementary data**

## Pharmacokinetic modelling

Initially, various mathematical functions were considered for pharmacokinetic modelling, including negative exponentials with or without a positive baseline (mathematical form: *f(t)* = *a∙e^(-b∙t)^*; *f(t)* = *a∙e^(-b∙t)^* + *d*), a sum of two negative exponentials (*f(t)* = *a∙e^(-b∙t)^* + *g∙e^(-h∙t)^*), and a single negative power function with and without a positive baseline (*f(t)* = *c_1_∙t^-c2^*; *f(t)* = *c_3_∙t^-c4^* + *c_5_*). A previous investigation (not reported in this work) evaluated the sensitivity of each function model to a few subsets of data points selected from a larger dataset of *FIA/g* derived with GC (e.g. subsets considering variations in the number of time points or in the number of mice per time point). In that investigation, from the functions considered, the negative power function resulted in the lowest variability in the estimation of the time-integrated dosimetry parameter from the different subsets of experimental data, and yet showed an *R*^2^ very similar to that of other (more complex) function models (e.g. difference in *R*^2^ of less than 0.004 compared to a bi-exponential model, for the subsets of data considered). Therefore, in this study a negative power function with two coefficients (*c_1_* and *c_2_*) (Equation s1) was chosen to model the time dependence of the kidney *FIA/g* of all datasets.

$FIA(t)/g\cong c_{1} t^{-c_{2}}$ *Eq. s1*

## SPECT activity calibration

To enable activity quantification from SPECT images, a calibration factor to convert voxel count rate (reconstructed “cps”) to voxel activity concentration (MBq.mL^-1^) was determined from scanning a 20-mL plastic syringe (18.6 mm internal diameter), filled with about 5 mL of [^131^I]-NaI aqueous solution with a calibrated activity concentration of at least 1.5 MBq.mL^-1^ determined by high-resolution gamma spectrometry analysis. A 30-min SPECT scan of the syringe (40 bed positions, 45 sec per bed position) was done. SPECT images were generated with the same reconstruction and correction settings used for mouse scans. A cylindrical volume of interest (VOI) was defined within the hot region on the reconstructed SPECT image, with a diameter equal to 75% of the syringe internal diameter and a length of 7 mm. The calibration factor was calculated as the ratio of the reference activity concentration of the ^131^I stock solution and the mean count rate of the VOI.

## Gamma counting measurements

The activity of dissected tissues was measured in a Cobra II model 5003 gamma counter (Canberra-Packard, Schwadorf, Austria) with a single 3-inch diameter NaI(Tl) through-hole detector. Each radioactive sample was measured for 30 seconds in a standard tube (8 mL plastic vials of 75 mm height and 12 mm diameter), using a fixed 260 to 470-keV photon energy window. The measurement protocol was optimized to limit the overall measurement uncertainty. Specifically, radioactive samples were measured when their activity was within the nearly linear response range (maximum -2.9% of non-linearity) and high enough as to record enough counts to achieve a counting statistical error within 2.0% expressed at a 95.5% CI (coverage factor *k*=2.0). Three rack sample positions were left empty between samples to reduce measurement cross-talk effects (which are significant for the gamma counter used) from activity in neighbouring samples to less than 0.1%. Measured count rates were corrected for remaining non-linearity using a second degree polynomial function experimentally derived in a previous investigation. Based on prior system performance tests, sample volume effects were estimated to be negligible (deviation within -0.3%) for all samples (sample volumes ≤ 0.5 mL). Background signal was subtracted from radioactivity measurements.

A unique calibration factor was used for all tissue samples to convert net count rate (“cpm”) to activity (kBq). The calibration factor was determined from 4 stock [^131^I]-NaI solutions, each one prepared and calibrated in an independent experiment but with same methods, to limit the uncertainty in GC measurements due to the reproducibility of the activity calibration procedure. A set of 3 (to 5) samples, consisting of standard tubes filled with 0.5 mL of solution with a calibrated activity in the range of 1 to 17 kBq, was prepared from each ^131^I stock solution. The reference activity values of the calibration samples were determined in 4 independent experiments (one for each stock solution) by high-resolution gamma spectrometry. Samples were counted using the same measurement protocol as tissue samples, and for each sample the measurement efficiency was calculated as the ratio of the sample reference activity and its net measured count rate. The overall calibration factor was calculated as the mean of the measurement efficiencies of the 4 stock solutions, each of which was calculated as the mean of the efficiencies of the set of samples.

## Reference measurements for SPECT and GC activity calibrations

The reference activity concentrations of all ^131^I stock solutions used for SPECT and gamma counting activity calibrations were determined by high-resolution gamma spectrometry analysis using a calibrated high-purity germanium detector (model GC1818-7500SL; Mirion-Canberra, Meriden, USA). From each reference stock solution, 3 (to 5) aliquots of 500 μL were dispensed in plastic vials of the same type as those used for gamma counting of dissected tissues. The content mass of each sample was verified gravimetrically. The photon energy spectrum of each sample was measured and the sample reference ^131^I activity was determined. Assuming a mass density of 1.00 g.mL^-1^, the solution activity concentration of each sample was determined from the sample reference activity divided by its mass content. Then, the reference activity concentration of the stock solution was determined as the average of the activity concentration of the samples set. The relative statistical uncertainty of the reference activity concentration of each of the calibration stock solutions was always within 1.6% at 95.5% confidence interval (coverage factor *k*=2).
